# Long-Term Outcomes and Prognostic Factors of Superficial Esophageal Cancer in Patients Aged ≥ 65 Years

**DOI:** 10.3389/fmed.2021.722141

**Published:** 2022-01-18

**Authors:** Jin Won Chang, Da Hyun Jung, Cheal Wung Huh, Jun Chul Park, Sung Kwan Shin, Sang Kil Lee, Yong Chan Lee

**Affiliations:** ^1^Department of Internal Medicine, Yonsei University College of Medicine, Seoul, South Korea; ^2^Department of Internal Medicine, Yongin Severance Hospital, Yonsei University College of Medicine, Seoul, South Korea

**Keywords:** esophageal cancer, endoscopic submucosal dissection, surgical resection, elderly, prognostic factors

## Abstract

**Background:**

The number of elderly patients with superficial esophageal cancer (SEC) is increasing. We aimed to evaluate the clinical outcomes and prognostic factors of overall survival (OS) in elderly patients undergoing endoscopic submucosal dissection (ESD) or surgical resection for SEC.

**Methods:**

Between January 2001 and May 2020, 290 patients aged ≥65 years who underwent ESD or surgical resection for SEC were evaluated. Their clinical outcomes and prognosis were assessed, and independent risk factors for OS were identified.

**Results:**

The mean patient age (269 men and 21 women) was 70.9 years (range 65–90 years). En bloc, R0, and curative resections were achieved in 94.5%, 90.0%, and 73.4% of the patients, respectively. During the follow-up [mean: 54.6 months (range: 1–210 months)], 79 patients died. The 3-, 5-, and 10-year OS rates were 82.5, 73.1, and 59.7%, respectively. In multivariate analysis, cancer history of the other organs, American Society of Anesthesiologists performance status, and presence of lymphovascular involvement (hazard ratio = 1.852, 1.656, and 1.943, respectively; all *P* < 0.05) were independent risk factors for poor OS. The high-risk group (≥2 risk factors) showed a significantly lower OS than the low-risk group (≤ 1 risk factor) (*P* < 0.001).

**Conclusions:**

The three risk factors could be useful in predicting the long-term prognosis of elderly patients with SEC.

## Introduction

Esophageal cancer is a major cause of cancer-related deaths worldwide and is the seventh most common malignant tumor (1). Approximately 300,000 patients die of esophageal cancer yearly worldwide (2). The 5-year survival rate is approximately 15–25%. The best results are related to early diagnosis (3); however, only 22% of superficial esophageal cancer (SEC) cases are detectable (4) mainly because SEC shows flat isochromatic features on conventional endoscopy (5), and most SEC or precancerous lesions show no symptoms (6). Recently, the use of Lugol chromoendoscopy and narrow band imaging has improved the detection of SEC (7, 8). With the recent increase in health check-ups, the number of elderly patients diagnosed with SEC is increasing (9).

SEC is a lesion in which tumor infiltration is limited to the basement membrane (Tis), mucosa (T1a), or submucosal layer (T1b) of the esophageal wall (10, 11). SEC can be treated with endoscopic submucosal dissection (ESD), surgical resection, and chemoradiation therapy (12). Surgical resection can be cured and has the advantage of knowledge of the exact stage, but it has high complication and morbidity. The outcome of chemoradiation therapy is favorable; however, it requires a long treatment period, and accurate histologic assessment is impossible (12). ESD is considered a curative treatment option for SEC in some cases, depending on the size, invasion depth, and extent of tumor (13). Therefore, the European Society of Gastrointestinal Endoscopy (ESGE) recommends ESD as the first-line treatment for superficial esophageal squamous cell carcinoma (14). ESD for SEC has a long-term survival equivalent to that of those treated with surgical resection (15). ESD cannot accurately determine whether lymph node metastasis is present; esophagectomy or chemoradiation therapy is therefore recommended when there is a risk of lymph node metastasis. Tumor histology, invasion depth, tumor differentiation, and lymphovascular invasion are known (13) risk factors for lymph node metastasis in SEC.

The life expectancy of western and eastern populations is increasing, and an increasing number of elderly patients are developing esophageal cancer (16). In the Republic of Korea (ROK), the average life expectancy in 2017 was 82.7 years (17); therefore, SEC treatment is important. In elderly patients, a different approach for SEC treatment may be needed because of comorbidities, poor general condition, and limited life expectancy related to aging. As the main cause of mortality in elderly patients with esophageal cancer is non-cancer-related death, survival and maintenance of a good quality of life are important in the management of elderly SEC patients. However, data on the long-term clinical outcomes of elderly SEC patients are relatively insufficient compared to younger patients (18–20). Therefore, we aimed to evaluate the clinical outcomes of elderly SEC patients and the prognostic factors for OS in long-term cohort data.

## Methods

### Patients and Study Design

We retrospectively reviewed the data of patients aged ≥65 years (21–23) who underwent ESD or surgical resection for SEC at Severance Hospital and Gangnam Severance Hospital between January 2001 and May 2020.

The inclusion criteria were as follows: (1) final pathology result was high-grade dysplasia, or squamous cell carcinoma; (2) tumor limited to the mucosa or submucosa; (3) age ≥ 65 years; and (4) treatment-naive esophageal cancer.

Patients who underwent neoadjuvant therapy (*n* = 27), patients without data confirming their survival or death (*n* = 21), and those with insufficient clinical or laboratory information (*n* = 4) were excluded.

SEC was defined based on the final histopathologic report of the resection specimen, since the tumor was limited to the mucosa or submucosa. ESD was performed for lesions that could be treated endoscopically according to the criteria of The Esophageal Cancer Practice Guidelines 2017 (24).

The study protocol was in accordance with the ethical guidelines of the 1975 Declaration of Helsinki and was approved by the Institutional Review Board of Severance Hospital (IRB number: 4-2020-1493). The requirement for informed consent was waived owing to the retrospective design of this study. All authors had access to the study data and reviewed and approved the final manuscript.

### Evaluation of Baseline Patient Characteristics

We assessed the patient's baseline demographic and clinical characteristics, including age, sex, presences of comorbidities, smoking history, alcohol history, cancer history of other organs and use of anticoagulant or antiplatelet medications. Presence of cancer history of other organs does not meant active cancer, but refers to a medical history that has been cured and is not currently receiving treatment.

We also evaluated possible prognostic factors, including the Onodera prognostic nutritional index (PNI) (25), neutrophil-to-lymphocyte ratio (NLR) (26), American Society of Anesthesiologist-performance status (ASA-PS) (27), and Charlson comorbidity index (CCI) (28). CCI was calculated as the sum of the scores assigned for several comorbidities based on the original definition (28). PNI and NLR were calculated based on blood sampling results.

### Histological Assessment

For pathologic specimen evaluation, tumor histology, grade of differentiation, tumor size, invasion depth, lymphovascular involvement (LVI), perineural involvement, and presence of tumor in the resection margin were evaluated. The definition of the histology assessment was based on the Japanese Classification of Esophageal Cancer, 11th Edition (11). T1a; Tumor invades mucosa, M1; Carcinoma *in situ*, M2; Tumor invades lamina proprima mucosae, M3; Tumor invades muscularis mucosae, T1b; Tumor invades submucosa (SM), SM1; Tumor invades the upper third of the submucosal layer, SM2; Tumor invades the middle third of the submucosal layer, SM3; Tumor invades the lower third of the submucosal layer.

### Follow-Up

Post-treatment surveillance for recurrence was performed. For the ESD and surgical resection groups, chest computed tomography (CT) was performed every 6 months for 2 years and annually thereafter for 5 years. Endoscopic evaluation was performed every 6 months for 2 years and annually thereafter for the ESD group. Annual endoscopic evaluation after treatment was performed in the surgical resection group. When cancer recurrence was detected, the patients underwent additional chest and abdominal CT scans.

### Short-Term Outcomes

Short-term outcomes were evaluated in terms of en bloc resection, R0 resection, curative resection, procedure time, duration of hospital stay, and adverse events. En bloc resection was defined as removal of the lesion in a single piece with a tumor-free margin. R0 resection was defined as en bloc resection with histopathological demonstration of horizontal and vertical margins free of cancer and dysplasia. Curative resection was defined as R0 resection without vascular invasion or lymph node metastasis on histology (29). When ESD is performed for SEC, En bloc resection is considered curative if the tumor is within the mucosa or invade the submucosa up to 200 μm without lymphovascular invasion (sm1) (30). In the ESD group, the procedure time was defined as the time from the start of dissection until the dissection was completed and the lesion was separated, and in the surgical resection group, it was defined as the time from the start of the incision to the time the suture was completed. Clinical signs of bleeding included hematemesis, melena, and hematochezia, and laboratory signs of bleeding were defined as a ≥ 2.0 g/dL decrease in hemoglobin level. Perforation was diagnosed radiologically using chest radiography or CT after the procedure. Pneumonia was diagnosed using chest radiography or CT after the procedure. Post-procedural stricture was defined as a stricture that required endoscopic treatment.

### Long-Term Outcomes

Long-term follow-up data were retrospectively collected from medical records. The all-cause mortality data of patients who did not regularly visit our institution were obtained from the National Health Insurance Corporation database. The date when SEC was first treated was defined as the index date, and the date of death from the index date was calculated. The relationship between OS and clinicopathological factors was evaluated. The clinicopathologic factors included patient characteristics (age, sex, smoking history, alcohol history, comorbidities, use of anticoagulants and/or antiplatelet drugs, PNI, NLR, ASA-PS, and CCI), lesion characteristics (tumor size, location, histologic type, gross appearance, circumferential spread, invasion depth, number of lesions, and presence of LVI), en bloc resection, R0 resection, and curative resection. OS was defined as the period from treatment to death from all causes. Follow-up periods were calculated from the date of ESD or surgery.

### Statistical Analysis

The patient's demographic, pathologic, and short-term clinical outcome data are summarized as the mean (minimum–maximum) for continuous variables and as numbers with percentages for categorical variables. OS was calculated using the Kaplan–Meier method and compared using the log-rank test. The OS was measured from the index date to the date of death or final follow-up, or the latest confirmation of survival. The relationship between OS and clinicopathologic features of patients was assessed using univariate and multivariate analyses using the Cox proportional hazard model. Hazard ratios (HRs) and 95% confidence intervals (CI) were calculated. When performing subgroup analysis comparing ESD and surgical resection groups, propensity score matching was performed using age, gender, and ASA-PS. The cutoff values of PNI and NLR were the values that maximized the sum of sensitivity and specificity for OS in receiver operating characteristic curve analysis. The value of dividing high- and low-risk groups was based on the sum of sensitivity and specificity, when the correlation between the patient's number of risk factors and the OS was analyzed using the receiver operating characteristic curve. All statistical analyses were performed using the SPSS software version 25.0 (IBM Corp., Armonk, NY, USA). Statistical significance was set at *P* < 0.05.

## Results

### Patient Characteristics

Among the 342 patients aged ≥65 years who underwent ESD or surgical resection for SEC, 52 were excluded according to our established exclusion criteria. Hence, 290 patients were selected for statistical analysis (Supplementary Figure 1). Of the 290 patients, 116 (40%) patients underwent ESD procedures, while 174 (93 laparoscopic esophagectomy and 81 open esophagectomy) patients underwent surgical resection.

Baseline characteristics of the study population are presented in Table 1. The comparison of baseline characteristics of patients who underwent ESD and those who underwent surgical resection is summarized in Supplementary Table 1. Patients who underwent ESD were significantly older; had higher BMI, serum creatinine level, and neutrophil count; had higher proportions of heavy drinkers, cancer history of the other organs, and use of anticoagulants or antiplatelet drugs; and lower PNI, ASA-PS, CCI, WBC count, and hemoglobin level than those who underwent surgical resection (all *P* < 0.05).

**Table 1:** Baseline characteristics of the 290 patients aged ≥ 65 years with superficial esophageal cancer.

**Variables**	**Value**
	All (*n* = 290, 100%)
**Demographic variables**	
Age, years	70.9 (65–90)
Male gender	269 (92.8)
Body mass index, kg/m2	21.7 (12.6–37.1)
**Smoking history**	
Never smoking	103 (35.5)
Smoker	187 (64.5)
**Alcohol history**	
Never drink or social drinker	251 (86.6)
Heavy alcoholics	39 (13.4)
**Comorbidities (with overlap)**	
Hypertension	158 (54.5)
Cardiovascular disease	35 (12.1)
Kidney disease	23 (7.9)
Diabetes mellitus	88 (30.3)
Hepatitis	7 (2.4)
Cerebrovascular disease	4 (1.4)
Cancer history of the other organs	32 (11.0)
Use of anticoagulants and/or antiplatelet drugs	53 (18.3)
**Prognostic factors**	
Prognostic nutritional index (range)	52.2 (11.0–69.3)
Neutrophil to lymphocyte ratio (range)	2.5 (0.7–15.5)
**ASA-PS score**	
1	74 (25.5)
2	107 (36.9)
3	105 (36.2)
4	4 (1.4)
**Charlson comorbidity index**	
0	24 (8.3)
1	11 (3.8)
2	145 (50.0)
3	78 (26.9)
4	16 (5.5)
5	11 (3.8)
6	5 (1.7)
**Laboratory variables**	
WBC count, 10^6^/L	6879.2 (3230–14210)
Hemoglobin, g/dL	13.7 (8.8–17.8)
Neutrophil count, 10^6^/L	4088.0 (1610–12780)
Lymphocyte, 10^6^/L	14987.9 (197–4740)
Serum fasting glucose, mg/dL	115.8 (49–275)
Blood urea nitrogen, mg/dL	18.3 (5.3–199.0)
Serum creatinine, mg/dL	1.0 (0.4–10.9)
Serum albumin, g/dL	4.2 (2.3–5.1)

*Data are presented as mean (minimum-maximum) or number (%). ASA-PS, American society of anesthesiologist physical status; WBC, white blood cell*.

### Lesion Characteristics

The lesion characteristics of the 290 patients are shown in Table 2. Most superficial esophageal neoplasms (*n* = 252, 86.9%) were squamous cell carcinomas, and the remaining 38 patients (13.1%) were high grade dysplasia, and most (*n* = 281, 96.9%) occurred in the middle or lower esophagus. Flat lesions were the most common (*n* = 226, 78.0%), and most (*n* = 260, 89.7%) were single lesions. The mean tumor size was 22.5 mm (range: 2.0–100.0 mm), and 144 (49.7%) had invaded the submucosal layer. LVI was observed in 32 (11.0%) patients, and perineural involvement was observed in one patient (0.3%).

**Table 2:** Lesion characteristics of the 290 patients aged ≥ 65 years underwent endoscopic submucosal dissection (ESD) or surgical resection for superficial esophageal cancer.

**Variables**	**Value**			
	**All (*n* = 290, 100%)**	**ESD (*n* = 116, 40%)**	**Surgical resection (*n* = 174, 60%)**	***P*-value**
Location				0.623
Upper	9 (3.1)	5 (4.3)	4 (2.3)	
Middle	125 (43.1)	49 (42.2)	76 (43.7)	
Lower	156 (53.8)	62 (53.4)	94 (54.0)	
Number of lesion				0.135
1	260 (89.7)	99 (85.3)	161 (92.5)	
2	24 (8.3)	14 (12.1)	10 (5.7)	
3	6 (2.1)	3 (2.6)	3 (1.7)	
Macrosopic type				0.002
Ip	1 (0.3)	0 (0.0)	1 (0.6)	
Is	32 (11.0)	6 (5.2)	26 (14.9)	
IIa	49 (16.9)	20 (17.2)	29 (16.7)	
IIb	157 (54.1)	78 (67.2)	79 (45.4)	
IIc	20 (6.9)	5 (4.3)	15 (8.6)	
III	31 (10.7)	7 (6.0)	24 (13.8)	
Elevated/Flat/Depression				0.012
Elevated	82 (28.2)	26 (22.4)	56 (32.2)	
Flat	157 (54.1)	78 (67.2)	79 (45.4)	
Depression	51 (10.7)	12 (10.3)	39 (22.4)	
Circumferential spread				<0.001
<25%	74 (25.5)	52 (44.8)	22 (12.6)	
25–50%	115 (39.7)	45 (38.8)	70 (40.0.2)	
50–75%	51 (17.6)	16 (13.8)	35 (20.1)	
≥75%	50 (17.2)	3 (2.6)	47 (27.0)	
Tumor size (mm), median
Tumor size	22.5 (2.0–100.0)	15.1 (2–48)	27.5 (2–100)	<0.001
Tumor depth				<0.001
Mucosa	146 (50.3)	89 (76.7)	57 (32.8)	
M1	22 (7.6)	11 (9.5)	11 (6.3)	
M2	93 (32.1)	59 (50.9)	34 (19.5)	
M3	31 (10.7)	19 (16.4)	12 (6.9	
Submucosa	144 (49.7)	27 (23.3)	117 (67.2)	
SM1	22 (7.6)	6 (5.2)	16 (9.2)	
SM2	94 (32.4)	18 (15.5)	76 (43.7)	
SM3	28 (9.7)	3 (2.6)	25 (14.4)	
Histologic type				<0.001
High grade dysplasia	38 (13.1)	26 (22.4)	12 (6.9)	
Well differentiated squamous cell carcinoma	93 (32.1)	49 (42.2)	44 (25.3)	
Moderate differentiated squamous cell carcinoma	136 (46.9)	38 (32.8)	98 (56.3)	
Poorly differentiated squamous cell carcinoma	23 (7.9)	3 (2.6)	20 (11.5)	
Lymphovascular involvement				0.066
Present	32 (11.0)	8 (6.9)	24 (13.8)	
Absent	258 (89.0)	108 (93.1)	150 (86.2)	
Perineural involvement				0.220
Present	1 (0.3)	1 (0.9)	0 (0.0)	
Absent	289 (99.7)	115 (99.1)	174 (100%)	
Lateral margin				<0.001
Positive	16 (5.5)	14 (12.1)	2 (1.1)	
Negative	274 (94.5)	102 (87.9)	172 (98.9)	
Vertical margin				0.048
Positive	2 (0.7)	2 (1.7)	0 (0.0)	
Negative	288 (99.3)	114 (98.3)	174 (100)	

*Data are presented as mean (minimum-maximum) or number (%). ESD, endoscopic submucosal dissection. P-value from comparison of ESD and surgical resection*.

The comparison of lesion characteristics of the 290 patients is summarized in Table 2. Patients who underwent ESD had more flat lesions, a higher proportion of circumferential spread of < 50%, higher proportion of positive resection margin, smaller tumor size, and less submucosal involvement than those who underwent surgical resection (all *P* < 0.05).

### Short-Term Outcomes

The short-term outcomes are shown in Table 3. In the 290 patients, the en bloc and R0 resection rates were 94.5% (*n* = 274) and 90.0% (*n* = 261), respectively. Curative resection was achieved in 213 (73.4%) patients. The adverse events were divided into those that occurred 48 h before ESD or surgical resection and those that occurred after 48 h of resection. Perforation occurred in six (2.1%) patients, all of whom underwent ESD, within 48 h after ESD or surgical resection. Adverse events that occurred after 48 h of ESD or surgical resection included bleeding (*n* = 3, 1.0%), perforation (*n* = 11, 3.8%), pneumonia (*n* = 42, 14.5%), and stricture (*n* = 54, 18.6%). Three (1.0%) patients in the surgical resection group died of adverse events. There were no ESD-related deaths. The mean procedure time and duration of hospital stay were 268.2 min (range: 6–635 min) and 20.4 days (range: 3–165 days), respectively.

**Table 3:** Short-term clinical outcomes of endoscopic submucosal dissection (ESD) and surgical resection for the 290 elderly patients with superficial esophageal neoplasm.

**Variables**	**Value**			
	**All**	**ESD (*n* = 116, 40%)**	**Surgical resection (*n* = 174, 60%)**	***P*-value**
En bloc resection	274 (94.5)	101 (87.1)	173 (99.4)	<0.001
R0 resection	261 (90.0)	90 (77.6)	171 (98.3)	<0.001
Curative resection	213 (73.4)	84 (72.4)	129 (74.1)	0.672
**Adverse event (<48 h)**				
Bleeding	0 (0.0)	0 (0.0)	0 (0.0)	-
Perforation	6 (2.1)	6 (5.2)	0 (0.0)	0.002
**Adverse event (>48 h)**				
Bleeding	3 (1.0)	2 (1.7)	1 (0.6)	0.343
Perforation	11 (3.8)	0 (0.0)	11 (6.3)	0.006
Pneumonia	42 (14.5)	3 (2.6)	39 (22.4)	<0.001
Stricture	54 (18.6)	16 (13.8)	38 (21.8)	0.085
Procedure time (minutes), median (range)	268.2 (6–635)	63.1 (6–352)	397.6 (81–635)	<0.001
Duration of hospital stay (days), median (range)	20.4 (3–165)	6.4 (3–31)	29.1 (4–165)	<0.001

*ESD, endoscopic submucosal dissection*.

### Long-Term Outcomes and Relationship Between OS and Clinicopathologic Factors

**Figure 1:**
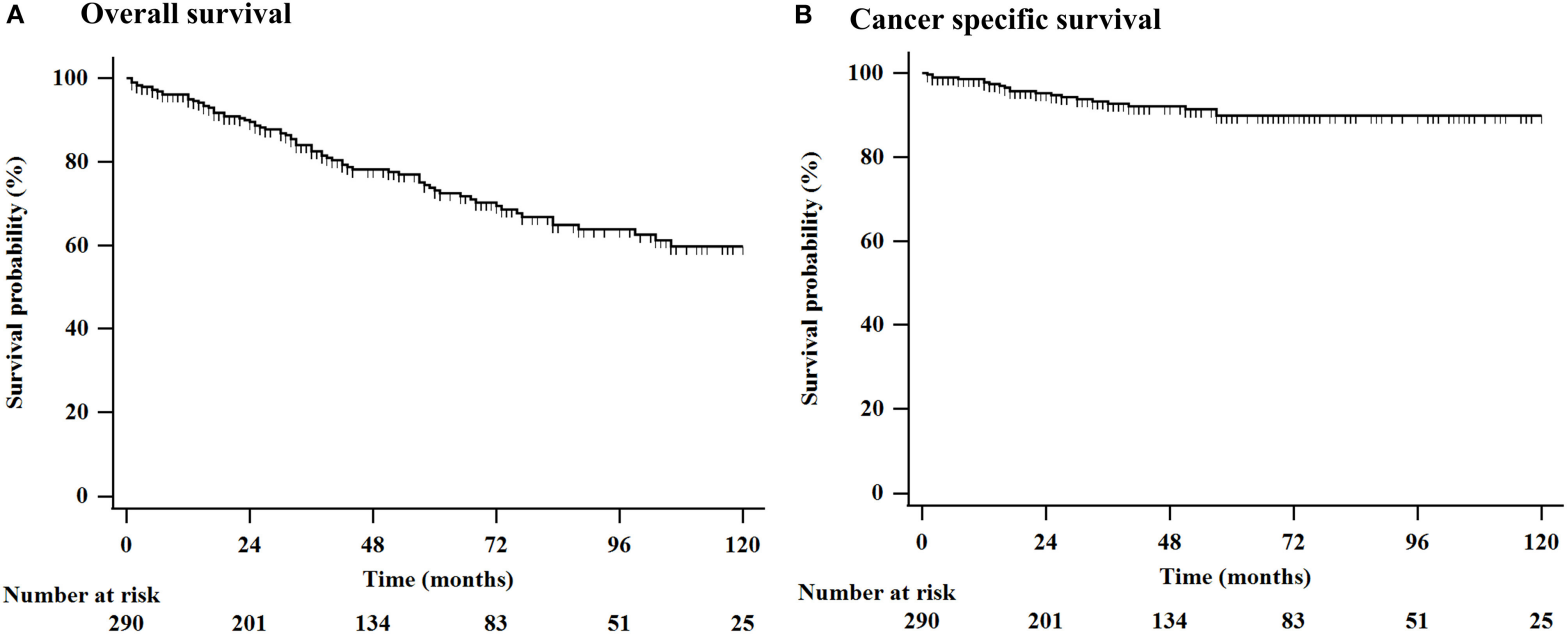
Kaplan-Meier estimation of OS in 290 elderly patients who underwent ESD or surgical resection for superficial esophageal cancer. **(A)** The 3-, 5-, and 10-year OS rates were 82.5, 73.1, and 59.7%, respectively, and **(B)** the 3-, 5-, and 10-year esophageal cancer related survival rates were 92.7, 89.8, and 89.8%, respectively. ESD, endoscopic submucosal dissection; OS, overall survival.

During the follow-up period (mean: 54.6 months, range: 1–210 months), a total of 79 (27.2%) patients died, 23 (7.9%) of whom died of esophageal cancer, and 56 (19.3%) died of other causes. Among the 77 (26.5%) patients who underwent non-curative resection, 29 (10.0%) underwent additional surgical resection, chemotherapy, or radiation therapy. Esophageal cancer recurred in 28 (9.7%) patients during the follow-up period. The mean period until recurrence was 52.8 months. For all patients, the 3-, 5-, and **7-**year OS rates were 82.5, 73.1, and 64.9%, respectively, and the 3-, 5-, and 7-year esophageal cancer-related survival rates were 92.7, 89.8, and 89.8%, respectively (Figure 1).

When the patients were divided into the ESD and surgical resection groups, the 3-, 5-, and 7-year OS rates in the ESD group were 87.0, 79.1, and 65.0%, respectively, and those in the surgical resection group were 78.7, 67.6, and 64.5%, respectively, with no statistically significant differences (*P* = 0.606) (Supplementary Figure 2). When analyzed except for patients who had been treated before 2010, the 3-, 5-, and **7-**year OS rates in the ESD group were 86.8, 78.9, and 64.3%, respectively, and those in the surgical resection group were 79.0, 66.6, and 64.1%, respectively, with no statistically significant differences (*P* = 0.562). After performing propensity score matching using age, sex, and ASA-PS, 107 patients were selected each when dividing the ESD and surgical resection group, the 3-, 5-, and 10-year OS rates in the ESD group were 88.0, 81.9, and 56.4%, respectively, and those in the surgical resection group were 80.3, 67.4, and 62.9%, respectively, with no statistically significant differences (*P* = 0.512).

When the patients were divided into the curative and non-curative resection groups, the 3-, 5-, and **7-**year OS rates in the curative resection group were 85.1, 75.8, and 66.9%, respectively, and those in the non-curative resection group were 74.0, 64.7, and 59.8%, respectively, with no statistically significant differences (*P* = 0.155) (Supplementary Figure 3).

The relationship between OS and clinicopathological factors is shown in Table 4. In univariate analysis, cancer history of the other organs, ASA-PS ≥ 3, and presence of LVI were associated with OS. In multivariate analysis, cancer history of the other organs (HR = 1.852; 95% CI, 1.010–3.397, *P* = 0.046), ASA-PS ≥3 (HR = 1.656; 95% CI, 1.012–2.710, *P* = 0.045), and presence of LVI (HR = 1.943; 95% CI, 1.004–3.762, *P* = 0.049) were independent risk factors for poor OS (Table 5). When calculating the number of risk factors, the correlation with OS through the receiver operating characteristic curve, the sum of sensitivity and specificity is the highest when divided by two. Patients with two or more of these risk factors and those with one or less of these risk factors were considered to be at “high risk” and “low risk,” respectively; accordingly, 268 (92.4%) and 22 (7.6%) patients were in the low-risk and high-risk groups, respectively. The high-risk group showed a significantly lower OS than the low-risk group (*P* < 0.001). In the low-risk group, the 3-, 5-, and **7-**year OS rates were 84.5, 76.3, and 67.6%, respectively. In the high-risk group, these rates were 51.8, 29.6, and 29.6%, respectively (Supplementary Figure 4).

**Table 4:** Relationship between overall survival and clinicopathologic factors.

**Clinicopathologic features**	**Number of patients**	**Number of death**	**3-year OS, % (95% CI)**	**5-year OS, % (95% CI)**	**7-year OS, % (95% CI)**	***P*-value**
**Patients characteristics**						
Age, years						0.061
<75	230	59	82.2 (76.7–87.7)	74.9 (68.2–81.6)	67.0 (59.2–74.8)	
≥75	60	20	83.8 (73.4–94.2)	65.0 (49.3–80.7)	54.5 (35.7–73.3)	
Sex						0.276
Male	269	75	81.8 (76.7–86.9)	72.3 (65.8–78.8)	63.5 (55.9–71.1)	
Female	21	4	90.5 (78.0–100.0)	82.3 (63.1–100.0)	82.3 (63.1–100.0)	
ESD or Surgical resection						0.606
ESD	116	35	87.0 (80.7–93.3)	79.1 (71.1–87.1)	65.0 (53.8–76.2)	
Surgical resection	174	44	78.7 (71.4–86.0)	67.6 (58.4–76.8)	64.5 (54.9–74.1)	
Smoking						0.474
Smoker	187	44	84.5 (78.8–90.2)	73.8 (66.0–81.6)	67.7 (58.5–76.9)	
Non-smoker	103	35	79.4 (70.5–87.7)	71.8 (61.−81.8)	61.0 (49.2–72.8)	
Alcohol						0.896
Heavy	39	10	88.0 (77.0–99.0)	81.2 (67.5–94.9)	54.3 (26.9–81.7)	
Non-alcohol, social	251	69	81.6 (76.3–86.9)	71.9 (65.3–78.6)	65.6 (58.0–73.2)	
Hypertension						0.146
Yes	158	43	81.6 (74.5–88.7)	68.3 (58.9–77.7)	57.8 (46.6–69.0)	
No	132	36	83.1 (76.2–90.0)	77.4 (69.4–85.4)	71.1 (61.7–80.5)	
Cardiovascular disease						0.803
Yes	35	10	86.8 (74.6–99.0)	77.6 (61.3–93.9)	61.7 (38.0–85.4)	
No	255	69	81.9 (76.6–87.2)	72.5 (65.8–79.2)	65.1 (57.5–72.7)	
Kidney disease						0.438
Yes	23	8	71.0 (51.4–90.6)	65.1 (43.9–86.3)	59.2 (37.1–81.3)	
No	267	71	83.6 (78.7–88.5)	73.8 (67.3–80.3)	65.4 (57.8–73.0)	
Diabetes mellitus						0.224
Yes	88	26	77.4 (67.4–87.4)	64.7 (52.2–77.0.2)	52.9 (38.2–67.6)	
No	202	53	84.6 (79.1–90.1)	76.6 (69.7–83.5)	69.7 (61.5–77.9)	
Cancer history of the other organs						0.002
Yes	32	16	71.7 (54.8–88.6)	60.1 (41.7–78.5)	43.0 (21.8–64.2)	
No	258	63	84.0 (79.1–88.9)	75.0 (68.5–81.5)	68.4 (60.8–76.0)	
Use of anticoagulants and/or antiplatelet drugs						0.773
Yes	53	17	87.3 (77.9–96.7)	75.4 (62.7–88.1)	66.5 (50.4–82.6)	
No	237	62	81.3 (75.6–87.0)	72.7 (65.8–79.6)	64.5 (56.3–72.7)	
Prognostic nutritional index						0.076
≤ 59.6	268	69	82.8 (77.7–87.9)	74.5 (68.2–80.8)	66.4 (59.0–73.8)	
>59.6	21	9	77.7 (58.3–97.1)	53.3 (26.6–80.0)	40.0 (9.8–70.2)	
Neutrophil to lymphocyte ratio						0.414
≤ 1.9	143	33	84.3 (77.6–91.0)	76.0 (67.2–84.8)	66.4 (55.4–77.4)	
>1.9	147	46	81.0 (74.1–87.9)	70.8 (62.4–79.2)	63.2 (53.4–73.0)	
ASA-PS						0.031
1 and 2	181	51	85.1 (79.4–90.8)	77.9 (71.0–84.8)	69.7 (61.5–77.9)	
3 and 4	109	28	77.5 (68.3–86.7)	61.4 (48.3–74.5)	53.8 (38.5–69.1)	
Charlson comorbidity index						0.594
≤ 2	180	49	83.5 (77.6–89.4)	75.5 (68.1–82.9)	68.0 (59.2–76.8)	
≥3	110	30	80.6 (72.2–89.0)	68.7 (57.7–79.7)	59.0 (46.1–71.9)	
**Lesion characteristics**						
Tumor size						0.251
<20 mm	146	38	85.2 (79.1–91.3)	78.1 (70.5–85.7)	66.9 (56.7–77.1)	
≥20 mm	143	40	79.1 (71.3–86.9)	66.7 (56.7–76.7)	61.6 (50.8–72.4)	
Histologic type						0.187
High grade dysplasia	38	8	85.2 (73.2–97.2)	77.9 (63.2–92.6)	77.9 (63.2–92.6)	
Cancer	252	71	82.0 (76.7–87.3)	72.2 (65.5–78.9)	62.6 (54.6–70.6)	
Lymphovascular involvement						0.021
Present	32	11	61.1 (39.7–82.5)	50.0 (27.7–72.3)	50.0 (27.7–72.3)	
Absent	258	68	84.5 (79.6–89.4)	75.4 (69.1–81.7)	66.3 (58.7–73.9)	
Curative resection						0.155
Curative	213	58	85.1 (79.8–90.4)	75.8 (68.9–82.7)	66.9 (58.5–75.3)	
Non–curative	77	21	74.0 (62.4–85.6)	64.7 (51.4–78.0)	59.8 (45.7–73.9)	
R0 resection						0.705
R0 resection	261	70	82.1 (76.8–87.4)	73.4 (66.9–79.9)	65.3 (57.7–72.9)	
Non_R0 resection	29	9	85.0 (71.3–98.7)	71.5 (53.3–89.7)	62.6 (39.9–85.3)	
En bloc resection						0.723
En bloc resection	274	74	82.7 (77.6–87.8)	72.6 (66.1–79.1)	64.8 (57.4–72.2)	
Piecemeal	16	5	79.3 (58.3–100.0)	79.3 (58.3–100.0)	63.5 (31.0–96.0)	
Location						0.208
Upper or mid	134	38	82.3 (74.9–89.7)	70.0 (60.2–79.8)	64.6 (54.6–74.6)	
Lower	156	41	82.3 (75.8–88.8)	75.2 (67.4–83.0)	68.5 (59.5–77.5)	
Gross appearance						0.566
Flat	163	40	85.1 (78.8–91.4)	74.9 (66.5–83.3)	59.0 (47.4–70.6)	
Non-flat	127	39	79.1 (71.5–86.7)	70.5 (61.5–79.5)	69.1 (60.1–78.1)	
Circumferential spread						0.502
<50%	189	49	83.2 (71.4–95.0)	76.3 (69.2–83.4)	65.8 (56.6–75.0)	
≥50%	101	30	81.3 (72.9–89.7)	67.3 (56.1–78.5)	62.8 (50.6–75.0)	
Depth of invasion						0.190
Mucosa	146	38	86 (79.9–92.1)	78.6 (71.0–86.2)	66.8 (56.8–76.8)	
Submucosa	144	41	78.3 (70.5–86.1)	66.2 (56.4–76.0)	62.4 (51.8–73.0)	
Number of lesion						0.088
1	260	68	83.7 (78.6–88.8)	74 (67.5–80.5)	66.1 (58.5–73.7)	
≥2	30	11	72.1 (54.3–89.9)	65.6 (45.2–86.0)	54.7 (28.8–80.6)	
Complication (Any)						0.005
Complication	59	20	69.6 (56.5–82.7)	51.8 (35.1–68.5)	67.2 (59.7–76.1)	
No complication	229	57	86.0 (80.9–91.1)	78.1 (71.6–84.6)	51.8 (35.1–68.5)	

*OS, overall survival; ESD, endoscopic submucosal dissection; ASA-PS, American society of anesthesiologist physical status*.

**Table 5:** Risk factors associated with poor overall survival.

**Variables**	**Univariate**		**Multivariate**	
	**HR (95% CI)**	***P*-value**	**Adjusted HR (95% CI)**	***P*-value**
**Patients characteristics**				
Age, years	1.067 (1.017–1.119)	0.008	1.050 (0.998–1.105)	0.057
Male gender	1.740 (0.634–4.779)	0.283		
Body mass index, kg/m2	0.963 (0.892–1.040)	0.332		
Smoking history	0.849 (0.540–1.332)	0.475		
Heavy alcoholics	0.956 (0.491–1.864)	0.896		
**Comorbidities (with overlap)**				
Hypertension	1.394 (0.889–2.185)	0.148		
Cardiovascular disease	1.088 (0.560–2.115)	0.804		
Kidney disease	1.336 (0.640–2.787)	0.441		
Diabetes mellitus	1.337 (0.835–2.140)	0.226		
Hepatitis	0.047 (0.000–19.486)	0.320		
Cerebrovascular disease	0.851 (0.118–6.135)	0.873		
Cancer history of the other organs	2.308 (1.320–4.035)	0.003	1.852 (1.010–3.397)	0.046
Use of anticoagulants and/or antiplatelet drugs	1.083 (0.631–1.859)	0.774		
**Prognostic factors**				
Prognostic nutritional index	0.996 (0.966–1.026)	0.772		
Neutrophil to lymphocyte ratio	1.004 (0.921–1.093)	0.936		
ASA PS score ≥3	1.699 (1.045–2.764)	0.033	1.656 (1.012–2.710)	0.045
Charlson comorbidity index ≥3	1.131 (0.718–1.783)	0.595		
**Lesion characteristics**				
Tumor size, mm	1.012 (0.995–1.030)	0.166		
Specimen size, mm	1.002 (0.998–1.006)	0.251		
Cancer (compared to high grade dysplasia)	1.627 (0.783–3.383)	0.192		
Lymphovascular invasion	2.097 (1.102–3.989)	0.024	1.943 (1.004–3.762)	0.049
Complete resection	0.696 (0.421–1.151)	0.158		
R0 resection	0.875 (0.436–1.754)	0.706		
En bloc resection	1.178 (0.474–2.927)	0.724		
Lesion location at lower esophagus	0.752 (0.481–1.175)	0.211		
Flat gross appearance	0.878 (0.564–1.369)	0.567		
Circumferential spread ≥ 1/2	1.169 (0.740–1.846)	0.503		
Depth of invasion (Submucosal invasion)	1.343 (0.862–2.093)	0.192		
Number of lesion	1.729 (0.913–3.276)	0.093		
Lateral margin	1.474 (0.639–3.401)	0.363		
Vertical margin	1.884 (0.261–13.610)	0.530		
Whole procedure time, minutes	1.000 (0.999–1.001)	0.825		
**Laboratory variables**				
WBC count, 10^6^/L	1.000 (1.000–1.000)	0.077		
Hemoglobin, g/dL	0.891 (0.773–1.027)	0.110		
Neutrophil count, 10^6^/L	1.000 (1.000–1.000)	0.303		
Lymphocyte, 10^6^/L	1.000 (1.000–1.001)	0.116		
Serum fasting glucose, mg/dL	1.001 (0.996–1.007)	0.638		
Blood urea nitrogen, mg/dL	0.989 (0.967–1.013)	0.367		
Serum creatinine, mg/dL	1.068 (0.850–1.342)	0.573		
Serum albumin, g/dL	0.642 (0.393–1.049)	0.077		

*HR, hazard ratio; ASA-PS, American society of anesthesiologist physical status; WBC, white blood cell*.

Twenty six (9.0%) of the total patients received additional treatment such as chemotherapy or radiotherapy. When logistic regression analysis was performed to evaluate whether additional treatment was a risk factor, the HR was 0.6 (95% CI, 0.222–1.681, *P* = 0.340), which was not statistically significant. When dividing patients who received and did not receive additional treatment, the 3-, 5-, and 7-year OS rate in the patients who received additional treatment were 80.0, 72.0, and 72.0%, respectively, and those in the patients who did not receive additional treatment were 82.7, 73.4, and 64.7%, respectively, with no statistical significant differences (*P* = 0.989).

### Subgroup Analysis-Comparison Between ESD and Surgical Resection

A comparison of short-term clinical outcomes is presented in Table 3. The en bloc and R0 resection rates were significantly higher in the surgical resection group (87.1 vs. 99.4%, 77.6 vs. 98.3%, respectively, *P* < 0.001). However, the curative resection rate was 72.4% in the ESD group and 74.1% in the surgical resection group, with no statistically significant difference (*P* = 0.672). Perforation and pneumonia that occurred after 48 h of ESD or surgical resection were higher in the surgical resection group (0 vs. 11%, 2.6 vs. 22.4%, respectively, *P* < 0.05). The mean procedure time and duration of hospital stay were significantly shorter in the ESD group than in the surgical resection group (63.1 vs. 397.6 min, 6.4 vs. 29.1 days, respectively, *P* < 0.001).

## Discussion

Esophageal cancer has a poor prognosis and must be detected and treated early. ESD or surgical resection is performed to cure SEC, and complications after the procedure and long-term prognosis are important issues. Elderly patients have many comorbidities, poor physical status, and shorter life expectancy than younger patients. Therefore, knowing the long-term prognosis or prognostic factors may help determine the treatment plan. To analyze OS after ESD or surgical resection for SEC in elderly patients, a long observation period is mandatory. Our study had a mean observation period of 54.6 months (range: 1–210 months) for 290 patients, which is a significant advantage. We analyzed the long-term outcomes and prognostic factors in elderly patients who underwent ESD or surgical resection for SEC.

In elderly esophageal cancer patients, analysis is necessary because various factors can affect OS. Therefore, we identified the short- and long-term outcomes and prognostic factors of patients aged ≥ 65 years who underwent ESD or surgical resection for SEC. The short-term outcomes of en bloc resection (94.5 vs. 97.1%), R0 resection (90.0 vs. 92.0%), curative resection (71.0 vs. 73.0–90.5%), perforation (3.8 vs. 0.0–12.1%), bleeding (1.0 vs. 2.0%), and stricture (18.6 vs. 5.1–25.9%) were similar to those reported in a previous meta-analysis study (31). The five-year OS (73.1 vs. 87.3%) and disease-specific survival (89.8 vs. 97.7%) rates were lower than those reported in the previous meta-analysis study (mean age: 70.9 vs. 64–71 years) (31).

In our study, cancer history of the other organs, ASA-PS ≥ 3, and presence of LVI were independent prognostic factors in the elderly patients with SEC. In a previous study, CCI ≥ 2 was identified as a prognostic factor in patients with esophageal cancer (20); however, in this study, CCI was not a prognostic factor for esophageal cancer. CCI is a useful assessment tool for comorbidities and a prognostic factor (20, 32); however, it might be insufficient to reflect the functional status or general condition of elderly patients. We found statistically significant differences in the 3-, 5-, and **7**-year OS rates between the low-risk and high-risk groups, which may help predict the prognosis of patients.

We performed the subgroup analysis according to treatment methods in elderly patients with SEC. Compared to patients who underwent surgical resection for esophageal cancer, those who underwent ESD were older, more obese, had a history of other cancers, and tended to take anticoagulants or antiplatelet drugs. This difference is presumed to be because of the preference for ESD, if possible, for older patients or those with more comorbidities, as surgical resection is associated with the burden of general anesthesia.

Esophageal cancer lesions in patients who underwent surgery tended to have larger and deeper tumor depths than those in patients who underwent ESD. There was no statistically significant difference in the curative resection rate between ESD and surgical resection; however, late adverse events occurred more frequently and hospital stay and procedure time were longer in the surgical resection group. This difference may have occurred because clinicians tend to choose ESD for patients with favorable tumor characteristics (small lesion, well-differentiated type, and less deep invasion). However, in the long term, there was no statistically significant difference in OS between the ESD and surgical resection groups, even though there was a difference in the baseline or lesion characteristics. Therefore, clinicians can select a procedure based on the lesion or patient condition.

In a meta-analysis (31), the 5-year OS (86.4 vs. 81.8%) and disease-specific survival (97.5 vs. 94.1%) rates of ESD and surgical resection were similar. It is unclear whether ESD or surgical resection is appropriate for elderly SEC patients. However, surgical resection causes more adverse events in elderly patients than ESD. Therefore, it would be better to perform ESD in high-risk patients. If additional surgery is needed after ESD, chemoradiation therapy or follow-up without additional surgery can be considered for patients at high risk rather than surgery. Since it was not a direct comparative analysis, caution should be exercised during interpretation; however, the criteria for the high- and low-risk groups, identified in this study, may not be an absolute standard for determining a treatment plan for elderly patients, but can be used as reference data.

Our study has several clinical implications. Our study involved a large number of patients aged ≥ 65 years who underwent ESD or surgical resection for SEC. In addition, during the follow-up period, the sufficient number of mortality cases (*n* = 79, 27.2%) might support the reliability of our study. Long-term prognosis and survival analysis were possible because the follow-up period was long, and the mortality rate was not small. Moreover, the median follow-up period of 54.6 months (maximum 210 months) was sufficient to identify long-term outcomes. Finally, we classified patients into low-risk and high-risk groups based on the risk factors we identified. Based on these results, we provided data that can be used as a basis for decision making when there is a concern about whether to perform ESD or surgical resection for SEC in elderly patients.

Our study has some limitations. First, our study had a retrospective design and therefore might have been subject to a potential bias. Patients who underwent neoadjuvant therapy were excluded because it was difficult to determine whether they were SEC patients; however, selection bias may have occurred during this process. Among the elderly patients, those who chose treatments other than ESD or surgical resection due to various underlying diseases or individual circumstances were excluded. Therefore, selection bias may have occurred. Further prospective studies on the prognosis and risk factors of ESD or surgical resection in the elderly are needed to validate our results. Second, this study was not a multicenter and multinational study. It was conducted with patients from two academic teaching hospitals in the ROK. Patients included in this study may not represent the entire elderly population and do not represent all SEC patients. Third, patients who did not undergo ESD or surgical resection and were only followed-up without treatment after esophageal cancer diagnosis were excluded. Some elderly patients, diagnosed with cancer are only followed-up without treatment for various reasons; therefore, for an accurate comparison of clinical outcomes, it may be necessary to compare patients who were observed without treatment with those who underwent ESD or surgical resection. Fourth, the maximum follow-up period was 210 months in our study. There have been advancements in techniques and devices, which may have caused a difference in prognosis and outcomes. Surgical techniques and instruments, endoscopic accessories, hemostasis methods, and drugs have been developed, and the accumulation of the operator's experience may have influenced the outcomes. However, the results were not significantly different when analyzed except for data in the early 2000's, so it seems that the impact was not significant. Fifth, LVI is not a prognostic factor that can be identified before the procedure; it may be difficult for the high-risk group criteria presented in our study to be used to fully predict patient's prognosis before the procedure. Sixth, comparison with the group of young patients was not analyzed.

In conclusion, we found that a history of cancer in other organs, ASA-PS ≥ 3, and presence of LVI were independent risk factors for poor OS in elderly patients undergoing ESD or surgical resection for SEC. These risk factors could be useful in predicting the long-term prognosis of elderly patients with SEC.

## Synopsis

The number of elderly patients with superficial esophageal cancer (SEC) is increasing. Cancer history of the other organs, American Society of Anesthesiologists performance status, and lymphovascular involvement were independent risk factors for poor overall survival in elderly patients with SEC.

## Data Availability Statement

The original contributions presented in the study are included in the article/Supplementary Materials, further inquiries can be directed to the corresponding authors.

## Author Contributions

JC, DJ, and CH: in planning and conducting the study and drafting the manuscript. JC, DJ, CH, JP, SS, SL, and YL: collecting and interpreting data. DJ: guarantor of the article. All authors contributed to the article and approved the submitted version.

## Funding

This work was supported by the National Research Foundation of Korea (NRF) grant funded by the Korea government (MSIT) (No. 2020R1C1C1013775).

## Conflict of Interest

The authors declare that the research was conducted in the absence of any commercial or financial relationships that could be construed as a potential conflict of interest.

## Publisher's Note

All claims expressed in this article are solely those of the authors and do not necessarily represent those of their affiliated organizations, or those of the publisher, the editors and the reviewers. Any product that may be evaluated in this article, or claim that may be made by its manufacturer, is not guaranteed or endorsed by the publisher.
