# Supplementary material for: Long-Term Outcomes and Prognostic Factors of Superficial Esophageal Cancer in Patients Aged ≥ 65 Years
Source: Front Med (Lausanne). 2022 Jan 18;8:722141. doi: 10.3389/fmed.2021.722141 (PMC8804291; doi:10.3389/fmed.2021.722141)
Supplement: Supplementary file 1 [file Table_1.docx]

| **Supplementary table 1**. The comparison of baseline characteristics of the 290 patients aged ≥65 years underwent endoscopic submucosal dissection (ESD) or surgical resection for superficial esophageal cancer | | | |
| --- | --- | --- | --- |
| Variables | Value | | |
|  | ESD (n=116, 40%) | Surgical resection  (n=174, 60%) | *P*-value |
| *Demographic variables* |  |  |  |
| Age, years | 72.1 (65-90) | 70.1 (65-80) | <0.001 |
| Male gender | 108 (93.1) | 161 (92.5) | 0.853 |
| Body mass index, kg/m2 | 22.5 (13.8-32.8) | 21.1 (12.6-37.1) | 0.001 |
| Smoking history |  |  | 0.582 |
| Never smoking | 39 (33.6) | 64 (36.8) |  |
| Smoker | 77 (66.4) | 110 (63.2) |  |
| Alcohol history |  |  | <0.001 |
| Never drink or social drinker | 83 (71.6) | 168 (96.6) |  |
| Heavy alcoholics | 33 (28.4) | 6 (3.4) |  |
| Comorbidities (with overlap) |  |  |  |
| Hypertension | 62 (53.4) | 96 (55.2) | 0.773 |
| Cardiovascular disease | 15 (12.9) | 20 (11.5) | 0.713 |
| Kidney disease | 12 (10.3) | 11 (6.3) | 0.214 |
| Diabetes mellitus | 31 (26.7) | 57 (32.8) | 0.274 |
| Hepatitis | 4 (3.4) | 3 (1.7) | 0.349 |
| Cerebrovascular disease | 4 (3.4) | 0 (0.0) | 0.014 |
| Cancer history of the other organs | 31 (26.7) | 1 (0.6) | <0.001 |
| Use of anticoagulants and/or antiplatelet drugs | 36 (31.0) | 17 (9.8) | <0.001 |
| *Prognostic factors* |  |  |  |
| Prognostic nutritional index (range) | 51.3 (11.0-61.8) | 52.9 (31.2-69.3) | 0.041 |
| Neutrophil to lymphocyte ratio (range) | 2.3 (0.7-10.1) | 2.6 (0.8-15.5) | 0.350 |
| ASA-PS score |  |  | 0.016 |
| 1 | 36 (31.0) | 38 (21.8) |  |
| 2 | 41 (35.3) | 66 (37.9) |  |
| 3 | 35 (30.2) | 70 (40.2) |  |
| 4 | 4 (3.4) | 0 (0.0) |  |
| Charlson comorbidity index |  |  | 0.027 |
| 0 | 15 (12.9) | 9 (5.2) |  |
| 1 | 8 (6.9) | 3 (1.7) |  |
| 2 | 54 (46.6) | 91 (52.3) |  |
| 3 | 24 (20.7) | 54 (31.0) |  |
| 4 | 7 (6.0) | 9 (5.2) |  |
| 5 | 6 (5.2) | 5 (2.9) |  |
| 6 | 2 (1.7) | 3 (1.7) |  |
| *Laboratory variables* |  |  |  |
| WBC count, 10^6^/L | 6565.6 (3600-12190) | 7086.5 (3230-14210) | 0.016 |
| Hemoglobin, g/dL | 13.4 (8.8-17.8) | 13.9 (8.9-17.8) | 0.001 |
| Neutrophil count, 10^6^/L | 3842.1 (1732-8330) | 2034.2 (197-4740) | 0.026 |
| Lymphocyte, 10^6^/L | 1918.2 (270-4200) | 2034.2 (197-4740) | 0.171 |
| Serum fasting glucose, mg/dL | 119.9 (49-275) | 113.1 (54-274) | 0.154 |
| Blood urea nitrogen, mg/dL | 21.1 (5.5-199.0) | 16.5 (6.8-32.6) | 0.057 |
| Serum creatinine, mg/dL | 1.1 (0.6-10.9) | 0.9 (0.4-1.8) | 0.047 |
| Serum albumin, g/dL | 4.2 (2.6-5.0) | 4.3 (2.3-5.1) | 0.175 |
| Data are presented as mean (minimum-maximum) or number (%). ASA-PS, American society of anesthesiologist physical status; WBC, white blood cell | | | |
