# Supplementary material for: Long-Term Outcomes and Prognostic Factors of Superficial Esophageal Cancer in Patients Aged ≥ 65 Years
Source: Front Med (Lausanne). 2022 Jan 18;8:722141. doi: 10.3389/fmed.2021.722141 (PMC8804291; doi:10.3389/fmed.2021.722141)
Supplement: Supplementary file 2 [file Presentation_1.pdf]

Supplementary figure legends

Supplementary Figure 1. Flowchart for selecting the study population. Among the 342 patients (≥65 years old) who underwent ESD or surgical resection for treatment-naive superficial esophageal cancer or high-grade dysplasia at the Severance Hospital and Gangnam Severance Hospital, between January 2001 and May 2020, 290 were selected for statistical analysis according to our inclusion and exclusion criteria. ESD, endoscopic submucosal dissection.

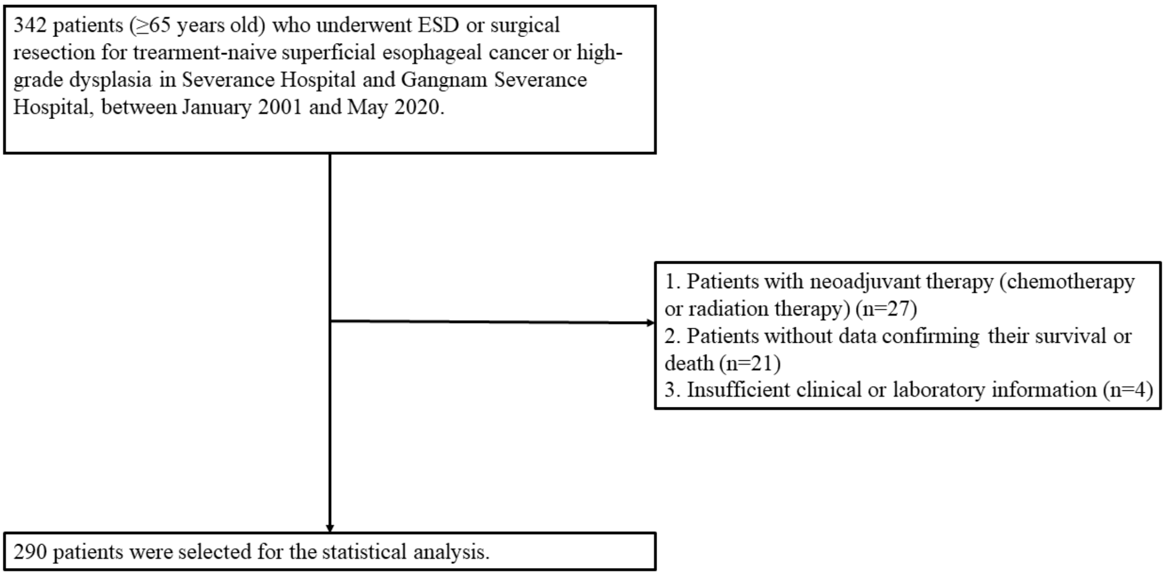

Supplementary Figure 2. Kaplan-Meier estimation of OS for each group. The 3-, 5-, and 10-year OS rates in the ESD group were 87.0%, 79.1%, and 52.9%, respectively, and those in the surgical resection group were 78.7%, 67.6%, and 64.5%, respectively (*P*= 0.606). OS, overall survival; ESD, endoscopic submucosal dissection.

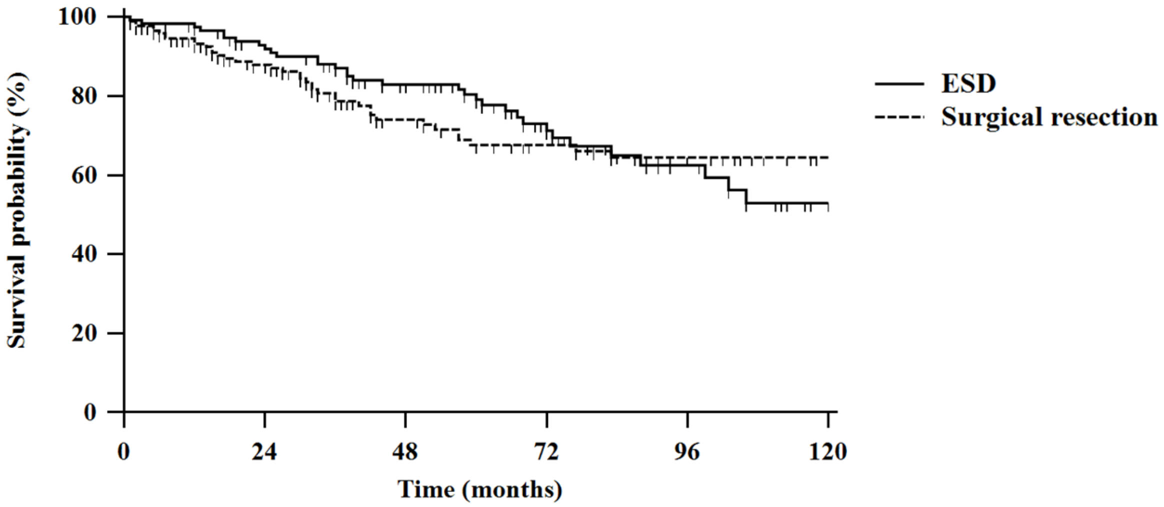

Number at risk

	0	24	48	72	96	120
ESD	116	96	73	39	21	7
Surgical resection	174	105	61	44	30	18

Supplementary Figure 3. Kaplan-Meier estimation of OS for each group. The 3-, 5-, and 10-year OS rates for the curative resection group were 85.9%, 76.4%, and 62.1%, respectively, and those for the non-curative resection group were 74.0%, 64.7%, and 53.1%, respectively (P= 0.155). OS, overall survival.

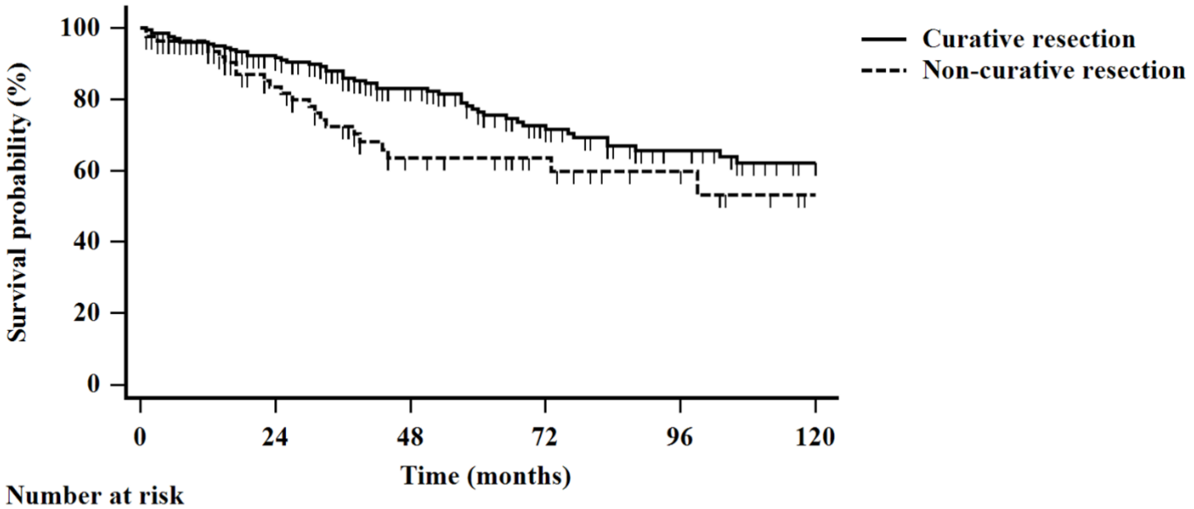

Number at risk						
Curative resection						
	206	154	108	66	42	22
Non-curative resection						
	84	47	26	17	9	3

Supplementary Figure 4. Kaplan-Meier estimation of OS for each group. The 3-, 5-, and 10-year OS rates in the low-risk group were 84.5%, 76.3%, and 62.2%, respectively and those in the high-risk group were 51.8%, 29.6%, and 29.6 %, respectively.

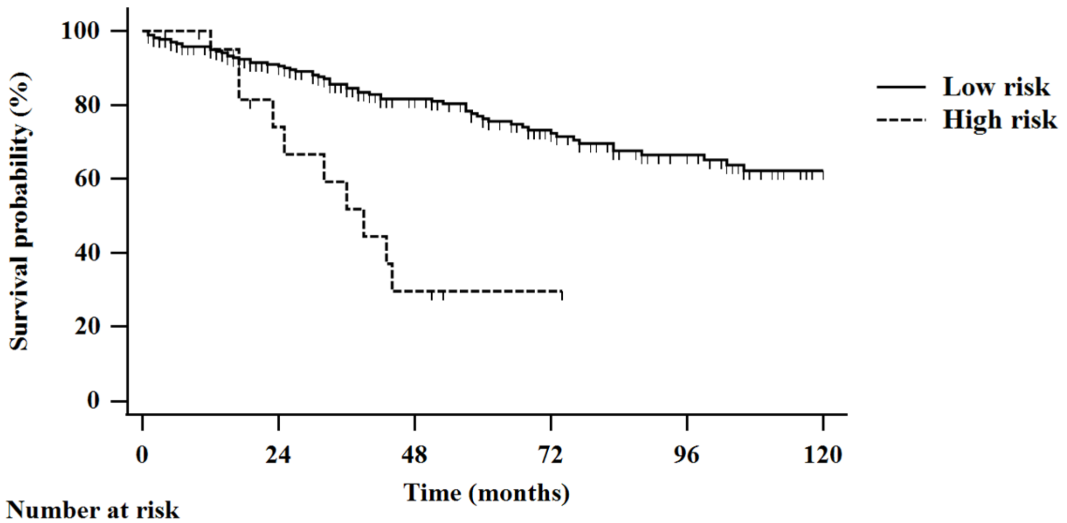

Number at risk

	0	24	48	72	96	120
Low risk	268	191	130	81	51	25
High risk	22	10	4	2	0	0
